# Cirsimaritin inhibits influenza A virus replication by downregulating the NF-κB signal transduction pathway

**DOI:** 10.1186/s12985-018-0995-6

**Published:** 2018-05-21

**Authors:** Haiyan Yan, Huiqiang Wang, Linlin Ma, Xueping Ma, Jinqiu Yin, Shuo Wu, Hua Huang, Yuhuan Li

**Affiliations:** 1 Beijing Key Laboratory of Antimicrobial Agents, Institute of Medicinal Biotechnology, Chinese Academy of Medical Sciences and Peking Union Medical College, Beijing, 100050 China; 2 Key Laboratory of molecular imaging of Shanghai Education Commission, Shanghai University of Medicine & Health Sciences, Shanghai, China; 3 Xinjiang Institute of Materia Medica, Urumqi, 830002 China

**Keywords:** Influenza a virus, Cirsimaritin, Antiviral activity

## Abstract

**Background:**

*Artemisia scoparia* Waldst and Kit is a famous traditional Chinese medicine widely distributed in Xinjiang, China. Flavonoids extracted from it exhibits inhibitory activities against several influenza virus strains. Despite this fact, the antiviral properties of CST, one of such flavonoids, against the influenza virus has not been reported. Thus, the aim of this study is to investigate the anti-influenza virus efficacy and antiviral mechanism of CST.

**Methods:**

The inhibitory activity of CST against influenza viruses was assessed by using viral titers and performing Western blot, qRT-PCR, and immunofluorescence assays in Madin–Darby canine kidney (MDCK) cells and a human monocytic cell line (THP-1). The mechanism of CST against influenza virus was analyzed by hemagglutination inhibition (HI) assay, neuraminidase (NA) inhibition assay, and Western blot.

**Results:**

CST reduced viral titers and influenza A virus (IAV) RNA and protein synthesis in a dose-dependent manner. Mechanistically, CST had no inhibitory effect on the attachment and release processes of the viral life cycle, as indicated by the HI and NA assays. Conversely, the CST-mediated inhibition of IAV is possibly linked to the inactivation of the NF-κB/p65 signal pathway. CST also suppressed the activation of JNK MAPK and P38 MAPK in vitro. In line with NF-κB/p65 inhibition, the expression levels of proinflammatory cytokines (TNF-α, IL-1β, IL-8, and IL-10) and the inflammation-related protein COX-2 were downregulated by CST.

**Conclusions:**

CST inhibited IAV replication by downregulating the NF-κB signal transduction pathway. CST may be a potential agent or supplement against IAV infection.

## Background

The influenza virus is a human respiratory pathogen with worldwide pandemic outbreaks. Meanwhile, the influenza A virus (IAV) is responsible for seasonal flu epidemic, which can lead to significant morbidity and mortality each year. IAVs is divided into various subtypes, such as H1N1 and H3N2, depending on their envelope glycoproteins. Notably, newly emerged influenza viruses, such as H7N9, H10N8, and H5N1, severely threaten human health. Notably, H7N9 causes severe illness and high death rates in patients [1]. Current anti-IAV drugs are mainly directed against the viral M2 protein (adamantane and rimantadine) and neuraminidase (NA; zanamivir, oseltamivir, and peramivir) [1, 2]. Although these drugs are effective against IAV replication, their efficacies decrease owing to the rapid emergence of drug-resistant viral mutants [3–5]. Hence, novel antiviral drugs against influenza virus are necessary. Targeting host factors, which are essential to influenza virus replication, is a good strategy for discovering novel antiviral drugs.

Nuclear factor kappa B (NF-κB) is a nuclear transcription factor widely present in various cells. NF-κB regulates over 100 target genes, such as those of cytokines, chemokines, growth factors, adhesion molecules, and antigen-presenting proteins. Most of these genes are involved in host immune and inflammatory responses. IAV infection activates the NF-κB pathway, thereby causing the overexpression of viral proteins, such as hemagglutinin (HA), NP, and M1 during viral infections [6–9]. Wurzer found that activating the NF-κB signaling pathway promoted the production of influenza viruses [10]. Furthermore, Kumar showed that inhibiting the NF-κB pathway by Bay 11-7082 and ammonium pyrrolidinedithiocarbamate restricts an early post-entry step during viral infection [11]. Similarly, cells with low NF-κB activity are virtually resistant to IAV infection. These findings suggest that influenza virus infection can be blocked by inactivating the NF-κB signaling pathway [12].

In China, traditional Chinese medicine has been used for the treatment of influenza virus infection for many years [13, 14]. *Artemisia scoparia* Waldst and Kit is widely distributed in Xinjiang, China and commonly used as a Uighur medicine, which has been used for preventing and treating cough, cold, and fever [15]. Flavonoids extracted from *A. scoparia* have inhibitory activities against influenza viruses [16]. Despite this fact, the antiviral study of cirsimaritin (CST), one of such flavonoids, has not been reported so far. In the present study, we demonstrated that CST (PubChem CID: 188323, Fig. 1a) inhibited the IAV. Interestingly, we found that IAV replication is inhibited after the downregulation of the NF-κB signal transduction pathway by CST.

**Fig. 1:**
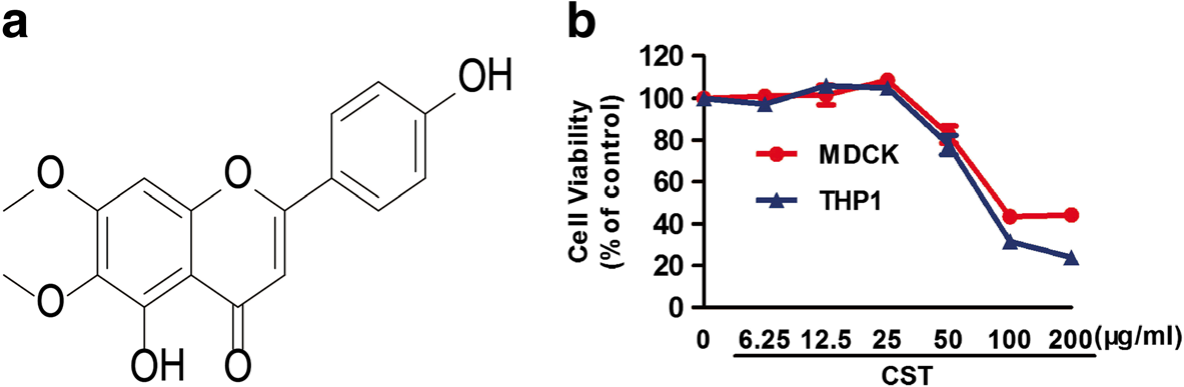
Chemical structure of CST and the cytotoxicity of CST. **a** The Chemical structure of CST. **b** Cytotoxicity of CST in MDCK and THP-1 cells under CCK assay. Different concentrations of CST were added to MDCK or THP-1 cells and incubated for 48 h. Cell viability was determined by CCK-8 assay. Data are expressed as mean ± SD, and the results represent the average findings of three independent experiments

## Results

### Cytotoxicity and antiviral activity of CST in vitro

To determine the antiviral activity of CST, we initially studied the cytotoxicity of CST in MDCK and THP-1 cells by using the CCK assay. The results showed that concentrations of less than 25 μg/ml showed no cytotoxicity in the MDCK or THP-1 cells within an observation period of 48 h (Fig. 1b). CST concentration of 20 μg/ml was selected as the maximum concentration in the following antiviral assays.

The TC_50_ values of CST and positive control of RBV in MDCK cells calculated by a CCK assay. By CPE assay we measured the antiviral activity of CST against influenza viruses in MDCK cells (Table 1). It showed that CST efficiently inhibited several influenza A virus strains, including A/tianjinjinnan/15/2009(H1N1) and A/JiangXi/312/2006(H3N2). The IC50 values of CST ranged from 5.8 to 11.1 μg/ml.

**Table 1:** Antiviral activity of CST against influenza virus strains

		H1N1				H3N2	
		A/Fort Mon mouth /1/1947		A/Tianjin Jinnan /15/2009		A/JiangXi/ 312/2006	
	TC_50_	IC_50_ (μg/ml)	SI	IC_50_ (μg/ml)	SI	IC_50_ (μg/ml)	SI
CST	153.3	6.3 ± 1.4	24.3	5.8 ± .22	26.4	11.1 ± 1.1	13.8
RBV	> 200	3.4 ± 0.2	58.8	6.0 ± 0.7	33.3	8.9 ± 0.5	22.5

**Fig. 2:**
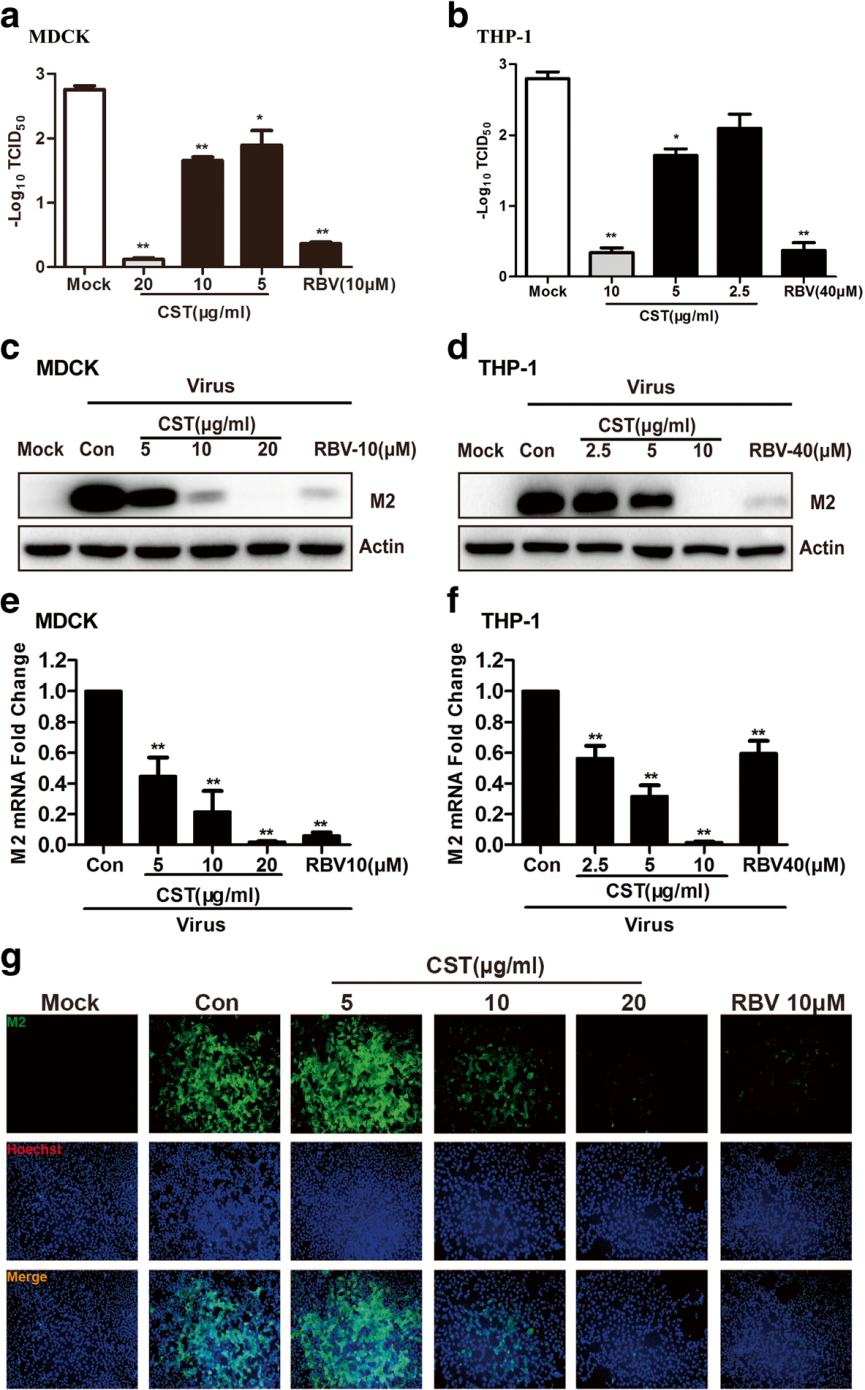
Antiviral effect of CST against A/Fort Monmouth/1/1947(H1N1). **a** and **b** MDCK and THP-1 cells were infected with A/Fort Monmouth/1/1947(H1N1) at 100TCID_50_, and CST was added after viral infection. RBV is the positive control. At 24 h post infection, cells were frozen and melt, and virus from the supernatant was harvested. Viral titers were quantified by cytopathic effect assay. Results were expressed as log_10_ values of the mean viral load ± SD. **c**, **d**, **e**, and **f** MDCK and THP-1 cells were infected with 100TCID_50_ influenza virus. The medium was changed 2 h after virus infection, and cells were treated with DMSO or CST. The cells were harvested 18 h after infection for Western blot or 12 h after infection for qRT-PCR. **p < 0.01 significantly different from the Con group. Data are expressed as mean ± SD, and the results represent the average of three independent experiments. **g** MDCK cells were infected with the 100TCID_50_ influenza virus. After infection for 2 h, MDCK cells were treated with CST or RBV for 18 h. The cells were stained with an anti-M2 antibody and imaged by immunofluorescence microscopy. M2 staining is shown in green. Nuclei stained by Hoechst are shown in blue

*TC*_*50*_ 50% toxicity concentration, *IC*_*50*_ 50% inhibitory concentration, *SI* selectivity index, SI = TC_50_/IC_50_;

The antiviral efficacy of CST was also tested by using viral titers. We observed a dose-dependent reduction in viral titers when the MDCK and THP-1 cells were treated with CST after infection (Fig. 2a and b). The results indicated that CST inhibited viral replication. In addition, we evaluated the inhibition ability of CST against the influenza virus in MDCK and THP-1 cells by Western blot and qRT-PCR. Our results showed that CST dose-dependently reduced the amounts of IAV M2 protein and RNA in MDCK cells (Fig. 2c and e). Similarly, CST showed a potent antiviral activity against IAV in the THP-1 cells, as reflected by the dose-dependent decrease in protein expression and M2 RNA (Fig. 2d and f). To further confirm that CST inhibited viral protein synthesis, we analyzed the expression of the viral M2 protein through indirect immunofluorescence assay. In Fig. 2g, CST exhibited a dose-dependent inhibition of the M2 protein expression in MDCK cells. Collectively, CST demonstrated a potent antiviral activity against the influenza virus.

### Non-inhibition by CST of viral hemagglutinin and NA functions

**Fig. 3:**
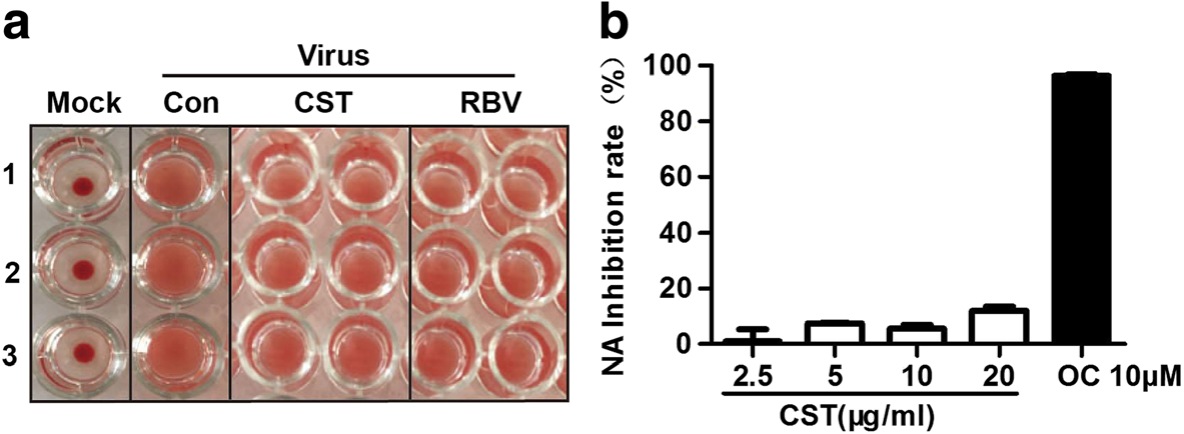
Inhibitory effect of CST on viral hemagglutination and NA. **a** Effects of CST on HI determined by chicken erythrocyte aggregation. **b** Effects of CST on IAV NA activity as examined by quantifying the fluorescent product upon 4-methylumbelliferyl- a-D-N-acetylneuraminic acid cleavage. Data are expressed as mean ± SD, and the results represent the average findings of three independent experiments

To elucidate the antiviral mechanism of CST, we initially determined whether CST restrained the function of the two IAV envelope glycoproteins (HA and NA), which are required for virus attachment and release [17]. However, the results showed that CST and RBV did not affect the hemagglutinin by the influenza virus (Fig. 3a). The findings also revealed that CST did not exert an inhibitory effect against NA, whereas OC, a known NA inhibitor, displayed significant inhibitory activity (Fig. 3b).

### CST-mediated inhibition of IAV replication by downregulating NF-κB expression

**Fig. 4:**
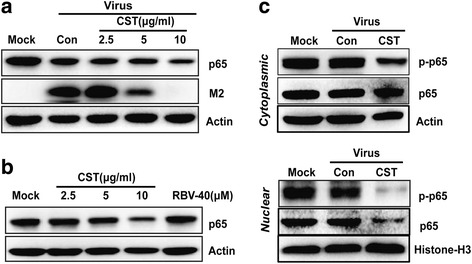
CST-mediated inhibition of influenza virus replication by reducing p65 expression and p65 phosphorylation. **a** THP-1 cells were infected with 100TCID_50_ IAV in the presence or absence of CST in different concentrations and RBV for 18 h. **b** THP-1 cells were treated with different amounts of CST and RBV for 18 h. **c** THP-1 cells were infected with 100TCID_50_ IAV and treated with 10 μg/ml CST for 30 min. The amount of cytoplasmic and nuclear p65 and phosphor-p65 protein were analyzed by Western blot

NF-κB is a nuclear transcription factor shown to play an important role in suppressing inflammation, oxidative stress, and host immunity [18]. In addition, accumulating evidence showed that downregulating NF-κB inhibits the replication of diverse species of viruses, including the influenza virus [11]. In this study, we found that CST dose-dependently decreased the level of intracellular p65/NF-κB protein in both infected and uninfected THP-1 cells (Fig. 4a and b).

To further determine whether CST disrupted IAV replication through the NF-κB signal pathway, we investigated the effects of CST on the phosphorylation of p65, a functional subunit of the NF-κB complex in the cytoplasm and nucleus. Initially, we found that CST inhibited the p65/NF-κB protein expression at 30 min of treatment in the nucleus. In addition, CST decreased the p65/NF-κB phosphorylation in the nucleus and cytoplasm (Fig. 4c). Collectively, our results showed that CST inhibits IAV infection by downregulating the NF-κB protein expression and inhibiting NF-κB phosphorylation in the nucleus.

### CST-mediated inhibition of the IAV-induced proinflammatory cytokine expression and COX-2 protein expression in THP-1cells

**Fig. 5:**
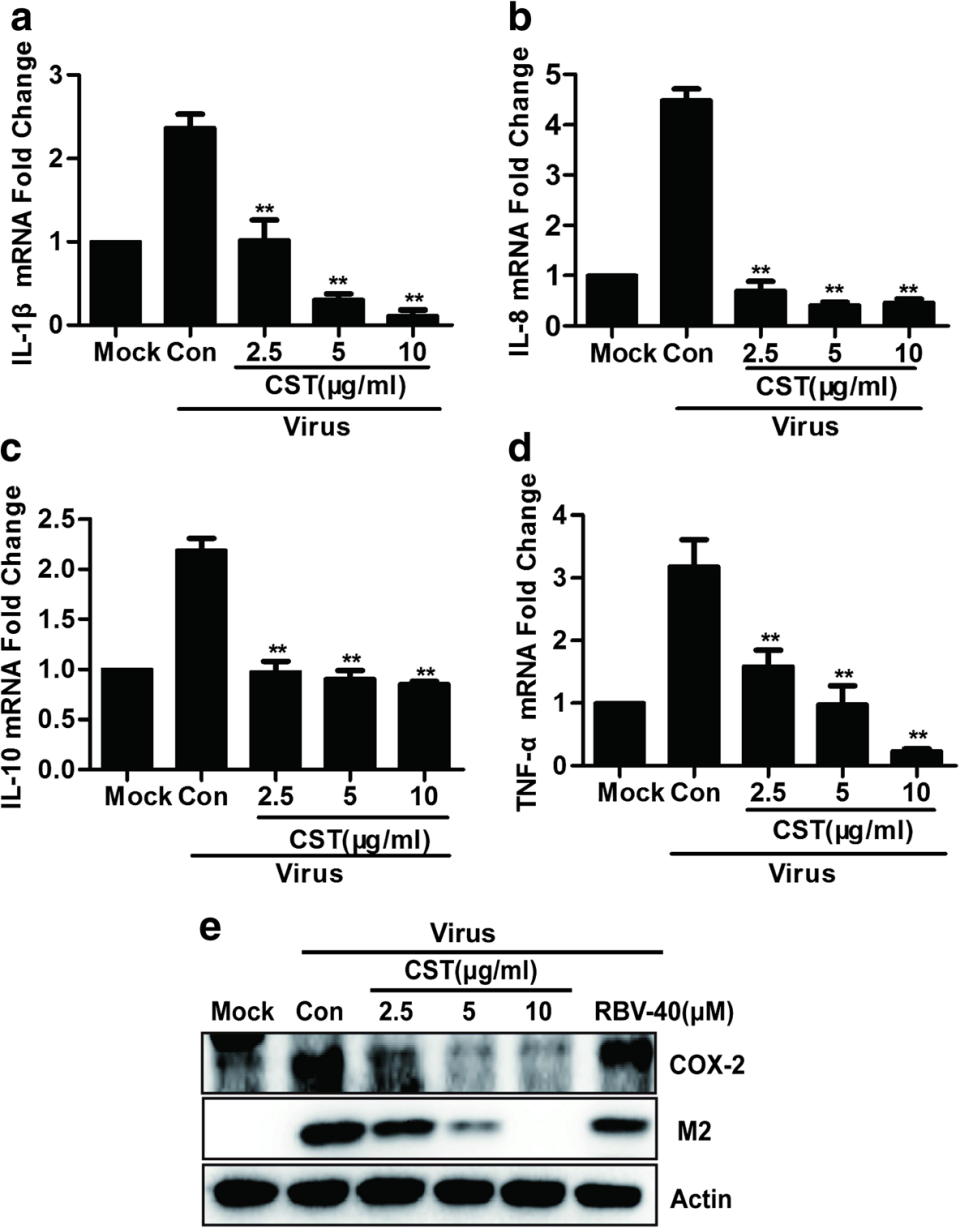
Effect of CST on the inhibition of proinflammatory cytokine production and COX-2 protein expression in THP-1 cells. **a**, **b**, **c** and **d** THP-1 cells were infected with 100TCID_50_ IAV and treated with 10 μg/ml of CST for 6 h. The IL-1β, TNF-α, IL-8, and IL-10 mRNA levels were determined by qRT-PCR. **p < 0.01 significantly different from the Con group. Data are expressed as mean ± SD, and the results represent the average findings of three independent experiments. **e** THP-1 cells were infected with 100TCID_50_ IAV in the presence or absence of CST of different concentrations and RBV for 18 h

NF-κB is a well-known central regulator of innate and adaptive immune responses. NF-κB also stimulates the expression of enzymes, such as COX-2 and various proinflammatory cytokines, which produce substances contributing to the pathogenesis of inflammatory processes [19, 20]. The H1N1-induced host cell expression of proinflammatory cytokines, including IL-8, IL-10, IL-1β, and TNF-α, has been correlated with disease in H1N1 and H5N1 patients [21]. Therefore, the inhibition of virus-induced cytokine release is also important in the treatment of influenza virus infections. We next examined the expression levels of IL-8, IL-10, IL-1β, and TNF-α in IAV-infected THP-1 cells regardless of the absence or presence of CST (Fig. 5a–d). Compared with the uninfected group, the H1N1-infected groups exhibited a dramatic increase (three folds) in the expression levels of IL-8, IL-10, IL-1β, and TNF-α. After treatment with CST, these cytokine levels were significantly reduced in a dose-dependent manner. In addition, we found that CST significantly reduced COX-2 protein expression in IAV-infected THP-1 cells (Fig. 5e).

### CST-induced suppression of the IAV-induced activation of MAPK signaling in the THP-1 cells

**Fig. 6:**
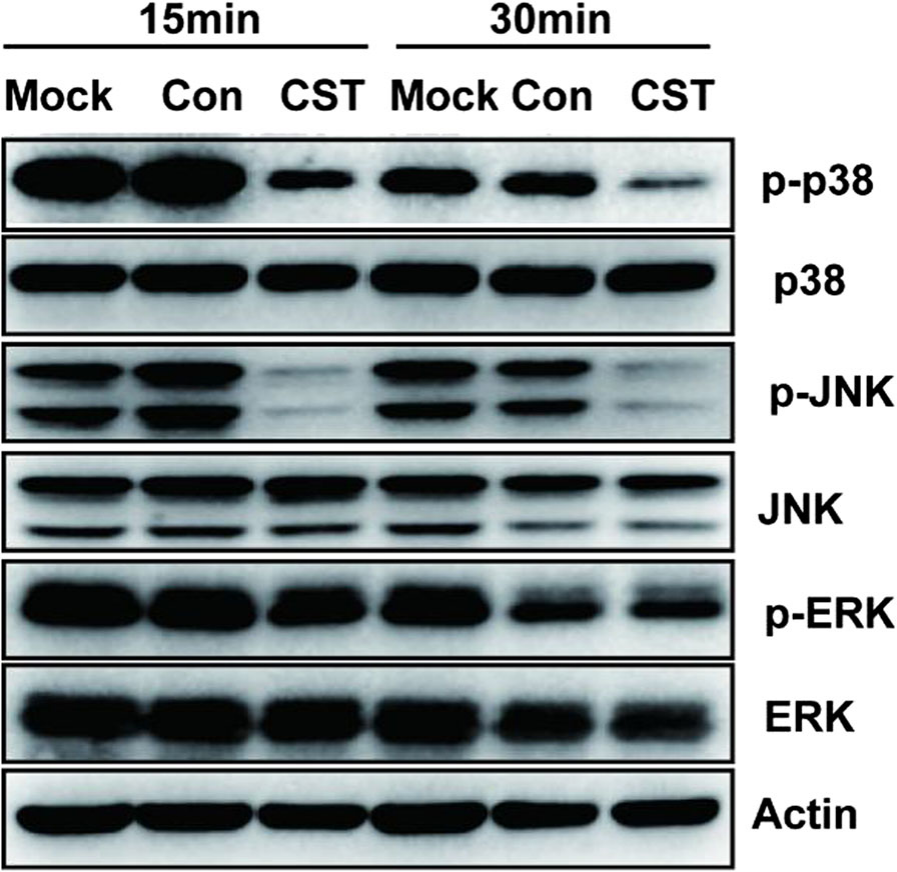
CST-mediated inhibition of p65 protein and p65 phosphorylation by reducing p38 MAPK and JNK phosphorylation. THP-1 cells were infected with 100TCID_50_ IAV and treated with 10 μg/ml of CST for 15 and 30 min. The phospho-p38 and phosphor-JNK proteins were analyzed by Western blot

To further explain the mechanism of CST’s inhibitory proinflammatory cytokine expression by CST, we then investigated the intervention of CST on the IAV-induced activation of MAPK signaling pathway. In our study, the phosphorylation of MAPKs was tested by lysing the treated THP-1 cells and determining the total proteins by Western blot. The protein levels of phospho-p38 MAPK and phospho-JNK remarkably decreased at 15 and 30 min, respectively, after the CST treatment (Fig. 6). On the contrary, CST showed no significant effect on ERK1/2 phosphorylation at these two time points.

## Discussion

Total flavonoids are considered as the main bioactive constituents of *A. scoparia*, which showed inhibitory activity against influenza viruses [16]. CST, an ingredient of the total flavonoids isolated from *A. scoparia*, is used as traditional Chinese medicine for anti-inflammatory, antioxidant, and antibacterial therapies [22–24]. To the best of our knowledge, this study is the first to demonstrate the anti-influenza virus activity of CST. The study results showed that CST inhibits the replication of IAV H1N1 and H3N2 in vitro.

To understand the mechanisms underlying the antiviral effect, we tested the effect of CST on influenza HA and NA protein in vitro and found that this compound did not inhibit the functions of these two IAV envelope glycoproteins but completely inhibited IAV replication. Moreover, CST did not inhibit the activity of RNA polymerase (data not shown). Therefore, the antiviral effect was most likely due to the inhibition of one or multiple intracellular replication events of the IAV.

A major host signaling pathway implicated in influenza virus replication is the NF-κB pathway. IAV infection has been shown to activate the NF-κB pathway [7]. In most cells, NF-κB exists as an inactive cytoplasmic complex, with the predominant form a heterodimer composed of p50 and p65 subunits, bound to inhibitory proteins of the IκB family. Following different stimulation types, such as the influenza virus, the NF-κB-IκB complex is activated by phosphorylating the inhibitory protein. This process then leads to the degradation of the inhibitory subunit and subsequent NF-κB release. The freed NF-κB dimers translocate to the nucleus, where the dimers activate the transcription of various genes involved in the inflammatory and immune response. In the nucleus, transcriptional activity control has been described to involve NF-κB phosphorylation. p65 phosphorylation by protein kinase A facilitates NF-κB association with the transcriptional coactivator CBP/p300 [25]. This occurrence potently enhances gene transactivation.

Monocyte–macrophages produce various cytokines, including IL-8, IL-10, IL-1β, and TNF-α, in response to NF-κB stimulation in the nucleus. The interaction of numerous inflammatory cytokines (TNF-α, IL-1β, and IL-8) and anti-inflammatory cytokines (IL-10) often lead to extensive pathological lung tissue damage. We observed that CST can reduce the level of p65 protein expression in THP-1 cells infected with IAV. In addition, CST downregulated nuclear p65 phosphorylation. Thus, the compound CST, which can reduce the production of TNF-α, IL-1β, IL-10, and IL-8 by inhibiting NF-κB can efficiently cure IAV infection.

In this study, CST significantly inhibited the phosphorylation of p38 and JNK, whereas ERK1/2 phosphorylation was unaffected (Fig. 6). Previous studies indicated that MAPKs, the upstream regulators of NF-κB, served a key role in LPS-induced inflammatory factor release from macrophages [26, 27]. Therefore, these CST effects on the IAV-mediated inactivation of JNK and p38 MAPK may also influence the NF-κB promoter activity. This result is supported by the CST of the IAV-induced downregulation of NF-κB protein expression and the inhibition of NF-κB phosphorylation in the nucleus. Hence, we suggest that the CST-induced downregulation of JNK and p38 MAPK phosphorylation negatively affects the IAV-induced increase in free NF-κB and p65 phosphorylation. This occurrence leads to the suppression of production of TNF-α, IL-1β, IL-10, IL-8, and COX-2, which are transcriptionally regulated by NF-κB.

## Conclusion

Our work provided a novel insight into the mechanisms of CST inhibited influenza A virus replication. We may conclude that CST had anti-IAV activity mainly through downregulating the phosphorylation of MAPKs (p38 and JNK) and NF-kB signaling pathway, regulating inflammatory cytokines such as IL-1β, IL-8, IL-10 and TNF-α. Taken together, our data demonstrated CST as a flavonoid compound, which provides a new prospect of CST for treating of influenza virus disease.

## Methods

### Cells and virus

The IAV A/Fort Monmouth/1/1947(H1N1) strain was obtained from the America Type Culture Collection (ATCC). A/tianjinjinnan/15/2009 (H1N1, oseltamivir- resistant), A/JiangXi/312/2006 (H3N2) were kindly provided by Professor Yuelong Shu at the Institute for Viral Disease Control and Prevention, China Centers for Disease Control and Prevention, Beijing, China. The viral stock of this strain was prepared using the method described previously [28].

Madin–Darby canine kidney cells (MDCK) were obtained from ATCC. MDCK cells were grown in minimum essential medium (MEM; Invitrogen, Carlsbad, CA, USA) supplemented with 1% MEM Non-Essential Amino Acid Solution (Invitrogen), 10% fetal bovine serum (FBS; Gibco, Grand Island, NY, USA), and 1% penicillin–streptomycin (10,000 U/mL) (Invitrogen). Human monocytic cells lines (THP-1) were obtained from National Infrastructure of Cell Line Resource, China. THP-1 cells were then cultured in RPMI-1640 Medium (Gibco, Grand Island, NY, USA) containing 10% FBS and antibiotics.

MDCK cell infection was performed as described previously [28]. For THP-1 cells, the maintenance medium was supplemented with 2% FBS and antibiotics.

### Compounds

CST and ribavirin (RBV) were purchased from Sigma–Aldrich (St. Louis, MO, USA). Oseltamivir carboxylate (OC) was purchased from Medchem Express (NJ, USA). CST (5 mg/ml) was dissolved in DMSO. Then, 2 mM stock solutions of RBV and OC were dissolved in the culture medium. These drugs were diluted to a final working concentration in the experiments.

### Cytotoxicity test

The cytotoxicity of CST in proliferating cells were assayed by the CCK method [29]. In brief, MDCK cells (2.5 × 104 cells per well) were seeded in 96-well plates overnight at 37 °C under 5% CO_2_. The media was removed, and serial two-fold dilutions of CST or control drugs were applied for 48 h. THP-1 cells (3 × 104 cells per well) were grown in 96-well plates, and then different concentrations of CST were added in the 96-well plates. After 48 h of incubation, 10 μl of CCK (Transgen, Beijing, China) was added to each well. After incubation at 37 °C for 2 h, the solution absorbance was measured at 450 nm on Enspire (Perkin Elmer, Waltham, MA, USA).

### Cytopathic effect (CPE) assays

MDCK cells (2.5 × 104 cells per well) were seeded in 96-well plates overnight at 37 °C under 5% CO_2_. The cells were infected with influenza virus (100TCID_50_) for 2 h, and then they were incubated with maintenance medium supplemented with or without CST. After further incubation for 48 h, the cytopathic effect were recorded. Then we calculated the 50% CPE inhibition concentrations (IC_50_) and the selectivity index (SI) values of CST.

### Hemagglutination inhibition (HI) assay

The inhibitory effect of CST on viral attachment was evaluated by the HI inhibition assay [28, 30]. Briefly, 50 μl of CST or RBV in serial two-fold dilutions in saline were mixed with an equal volume of influenza virus suspension and incubated for 30 min at 4 °C. Saline was used as positive control, and the no-virus red blood cell (RBC) was used as the negative control. Afterward, 100 μl of 1.2% chicken RBCs was dispensed to each well and incubated at room temperature for 40 min to monitor for agglutination.

### NA inhibition assay

By quantifying the fluorescent product upon the cleavage of 4-methylumbelliferyl- a-d-N-acetylneuraminic acid (MUNANA; Sigma, St Louis, MO, USA) by NA, IAV NA activity was assessed [28, 31]. The reaction system contains 20 μl of sample, 20 μl of enzyme and 60 μl of substrate buffer mix (20 μM MUNANA, 33 mM MES buffer (pH 3.5), 4 mM CaCl_2_, and double distilled water). Briefly, neuraminidases derived from influenza A viruses were incubated with diluted drug samples for 60 min at room temperature and then 60 μl neuraminidases substrate was added. The luminescence signal indicating neuraminidase activity was determined on Enspire (Perkin Elmer, Waltham, MA, USA) with excitation wavelength 355 nm and emission wavelength 460 nm before and after incubating for 15 min at 37 °C. The inhibition ratio was calculated according to the equation.

### Indirect immunofluorescence assay

MDCK cells (3 × 105 cells) grown on a coverslip in 12-well plates. The cells were infected with influenza virus (100TCID_50_) for 2 h with or without CST (5, 10, and 20 μg/ml) or RBV (10 μM). Cells were fixed with 4% paraformaldehyde for 10 min at room temperature at 18 h post-infection (p.i.). After incubating with 0.5% Triton X-100 for 15 min, cells were blocked with 1% bovine serum albumin (BSA) in PBS for 1 h at room temperature. The cells were then incubated with appropriate primary (IAV M2 antibody, Santa Cruz, Dallas, Texas, USA) and secondary antibodies (Alexa-Fluor-488, Transgen, Beijing, China). The nucleus was detected with Hoechst 33,342 (Beyotime, Shanghai, China). Pictures were taken with an Olympus TH4-200 microscope.

### Western blot

Cells were lysed in M-PER mammalian protein extraction reagent containing the Halt Protease inhibitor cocktail (Thermo Fisher Scientific, Waltham, MA, USA), whereas nuclear and cytosolic extracts were prepared using a nuclear and cytoplasmic extraction kit (Beyotime, Beijing, China). Lysates were centrifuged at 12,000 g for 15 min at 4 °C. The supernatants were collected and an equal amount of proteins were subjected to 10% SDS-PAGE. Proteins were detected using antibodies directed against ERK 1/2(1:1000), phosphorylated ERK 1/2 (1:1000), p38 (1:1000), phosphorylated p38 (1:1000), JNK (1:1000), phosphorylated JNK (1:1000), nuclear factor-kappa B subunit p65 (NF-κB p65) (1:1000), phosphorylated nuclear factor-kappa B subunit p65 (NF-κB p65) (1:1000), cyclooxygenase-2 (COX-2; 1:1000), β-actin (1:5000), histone H3 (1:1000) (Cell Signaling Technology, Beverly, MA, USA), IAV M2 (1:400) (Santa Cruz, Dallas, Texas, USA), respectively. Proteins were visualized by an ECL kit (GE Healthcare Life Sciences, Pittsburgh, PA, USA). Membranes were exposed to Biorad GelDoc XR (Bio-RAD, USA).

### Quantitative real-time PCR

MDCK and THP-1 cells were infected with 100TCID_50_ influenza virus for 2 h and cultured in the presence of CST at different concentrations. At 12 h p.i., total RNA was isolated from cells by using RNeasy Mini Kit (Qiagen, Germantown, MD, USA).

The primers used to amplify the IAV M2, IL-1β, TNF-α, IL-8, IL-10, and GAPDH genes (Table 2), were synthesized by Invitrogen Biotechnology Co. Ltd., China. The relative quantification of these genes were carried out with an ABI 7500 Fast real-time PCR instrument (Applied Biosystems, Foster City, CA, USA) by using TransScript II Green One-Step qRT-PCR SuperMix kit (TransGen Biotech, China) with the following procedures: 50 °C for 5 min, 94 °C for 30 s, followed by 35 cycles of 94 °C for 5 s, and 60 °C for 30 s. The relative mRNA amounts of IAV M2, IL-1β, TNF-α, IL-8, as well as IL-10, were then calculated by comparative Ct method after normalizing against the quantity of GAPDH.

**Table 2:** Oligonucleotides used for real-time RT-PCR

OLIGONUCLEOTIDE	SEQUENCE (5’–3’)
5’ M2 (INFLUENZA)	GACCRATCCTGTCACCTCTGAC
3’ M2 (INFLUENZA)	GGGCATTYTGGACAAAKCGTCTACG
5’ IL-1β	CTCGCCAGTGAAATGATGGCT
3’ IL-1β	GTCGGAGATTCGTAGCTGGAT
5’ TNF-α	ACTTTGGAGTGATCGGCC
3’ TNF-α	GCTTGAGGGTTTGCTACAAC
5’ IL-8	GGTGCAGTTTTGCCAAGGAG
3’ IL-8	TTCCTTGGGGTCCAGACAGA
5’ IL-10	AGGATCAGCTGGACAACTT
3’ IL-10	GATGTCTGGGTCTTGGTTCTC
5’ GAPDH (H)	GGTGGTCTCCTCTGACTTCAACA
3’ GAPDH (H)	GTTGCTGTAGCCAAATTCGTTGT
5’ GAPDH (D)	AGTCAAGGCTGAGAACGGGAAACT
3’ GAPDH (D)	TCCACAACATACTCAGCACCAGCA

### Statistics

Statistical analyses were performed by SPSS 19.0 software. All data are given as the mean ± standard deviation (SD). Two groups were compared by student’s-test, more groups were compared by one-way ANOVA. Differences with a *P* value of < 0.05 were considered statistically significant.

## Abbreviations

COX-2: Cyclooxygenase-2
CST: Cirsimaritin
HI: Hemagglutination inhibition
IAV: Influenza A virus
MDCK: Madin–Darby canine kidney cells
NA: Neuraminidase
NF-κB: Nuclear factor kappa B
OC: Oseltamivir carboxylate
RBV: Ribavirin
THP-1: Human monocytic cells lines
